# Supplementary material for: Triggered Ferroelectricity in HfO2 From Hybrid Phonons and Higher‐Order Dynamical Charges
Source: Adv Mater. 2026 Mar 10;38(19):e21602. doi: 10.1002/adma.202521602 (PMC13040540; doi:10.1002/adma.202521602)
Supplement: Supplementary file 1 — Supporting File: adma72722‐sup‐0001‐SuppMat.pdf. [file ADMA-38-e21602-s001.pdf]

Supporting Information for
Triggered ferroelectricity in HfO_2 from hybrid phonons and higher-order dynamical charges

Seongjoo Jung, Turan Birol*
*Corresponding author. Email: tbirol@umn.edu

Computational Method

$7 \times 7 \times 7$ Γ-centered **k**-point mesh was used to sample the Brillouin zone of the 12-atom supercell of fluorite HfO_2. Optimization of structures was converged below 10^{-7} eV of electronic energy changes and 1.0 meV/Å of maximum ionic forces. Fixed-ion calculations were converged below 10^{-6} eV of energy changes. Lattice parameter for cubic HfO_2 was 5.020 Å. Biaxial strain was simulated by fixing lattice vectors $\boldsymbol{a}$, $\boldsymbol{b}$ and allowing $\boldsymbol{c}$ to relax. (The η_{zz} strain has same irrep as energy for a parent structure I4/mmm, appearing implicit in the Landau equation [1]). Lattice parameters under 1% tensile strain were $a = b = 5.070$ Å, $c_0 = 5.002$ Å for I4mmm phase, $c = 5.038$ Å for Pca2_1 phase, $c = 5.112$ Å for P4_2/nmc phase and $c = 5.020$ Å for Aeaa phase. Polarization was calculated using the Berry phase definition from modern theory of polarization [2]. $15 \times 15 \times 15$ Γ-centered **k**-point mesh, and $160 \times 160 \times 160$ fine real space mesh was used for charge density representation of the 12-atom cubic supercell.

A 30-atom and a 48-atom supercell were used to simulate the ground state structures of $LiNbO_3$ and $Ca_3Ti_2O_7$, corresponding to the space groups R$\bar{3}$c (#161) and Cmc2_1 (#36), respectively. For $LiNbO_3$, a Γ-centered $8 \times 8 \times 3$ **k**-point mesh and a plane-wave kinetic energy cutoff of 650 eV were employed, while for $Ca_3Ti_2O_7$, a Γ-centered $6 \times 6 \times 2$ **k**-point mesh and a cutoff energy of 520 eV were used. The valence electron configurations adopted were $1s^2 2s^1$ for Li, $4s^2 4p^6 4d^4 5s^1$ for Nb, $3s^2 3p^6 4s^2$ for Ca, and $3s^2 3p^6 3d^3 4s^1$ for Ti. The optimized lattice parameters of the ground state structures were $a = b = 5.13$ Å, $c = 13.80$ Å, $\gamma = 120°$ for $LiNbO_3$; $a = 5.44$ Å, $b = 5.39$ Å, $c = 19.31$ Å for $Ca_3Ti_2O_7$; and $a = 5.20$ Å, $b = 5.00$ Å, $c = 5.02$ Å for the HfO_2 supercells. The space groups of each ground structures separated into polar and non-polar modes were R3m (#160) and R$\bar{3}$c (#167) for $LiNbO_3$, Fmm2 (#42) and Cmc2_1 (#36) for $Ca_3Ti_2O_7$, and Fmm2 (#42) and Pca2_1 (#21) for HfO_2.

Space group representations, mode definitions and couplings were referred from the Bilbao Crystallographic Server[3] and FINDSYM, ISODISTORT, and INVARIANTS from the ISOTROPY software suite[4–7]. All modes present in both the polar and nonpolar minima was be considered. (The condensed modes in the triggered phase also include those in the dielectric phase.) Additionally, we assume that no additional mode condenses exclusively between the two minima, which is a valid assumption for macroscopic modeling of ferroelectricity without domain separation where no net polarization should appear in the in-plane direction without electrode screening. The following order parameter definition involving normalization factor $\sqrt{N}$ was used, where N is the number of primitive cells in the u.c. and u is the displacement of an ion based on the lattice parameters of high-symmetry I4/mmm structure[8]:

$$|Q| = \sqrt{\frac{\Sigma {u_{i\alpha}}^2}{N}} \tag{S1}$$

i denotes iteration over each ion in the u.c., and α over each Cartesian directions.

The equation and coefficients used for the figures are as follows, which are unitless and chosen for illustrative purposes:
Fig. 2a, 3a: Equation (1),
$(\beta_0, \beta_1, \beta_2, \gamma, \delta_0, \delta_{12}, \lambda) = (0.3, 0.2, 0.1, 0.3, 1, 0.03, 1)$.
Fig. 2b: Equation (7),
$(\beta_0, \beta_1, \beta_2, \beta_3, \beta_4, \gamma_{012}, \gamma_{134}, \delta_0, \delta_{12}, \delta_{13}, \delta_4, \lambda) = (0.3, 0.2, 0.1, 0.1, -0.4, 0.3, 0.25, 1, 0.03, 0.12, 1.6, 1)$.
Fig. 3b:

$$H = \frac{\beta_0}{2}{p_0}^2 + \frac{\beta_1}{2}{q_1}^2 + \frac{\beta_2}{2}{q_2}^2 + \gamma p_0 q_1 q_2 + \frac{\delta_1}{4}{q_1}^4 + \frac{\delta_2}{4}{q_2}^4 + -\nu(\lambda p_0) \tag{S2}$$

$(\beta_0, \beta_1, \beta_2, \gamma, \delta_1, \delta_2, \lambda) = (0.05, -0.2, -0.1, 0.02, 0.1, 0.1, 1)$.
Fig. 5a: Equation (8),
$(\beta_0, \beta_1, \beta_2, \gamma, \delta_0, \delta_{12}, \lambda, \mu) = (0.3, 0.2, 0.1, 0.3, 1, 0.03, 1, 0.07)$.
Fig. S3: Equation (1), $(\beta_1, \beta_2, \delta_0, \delta_{12}, \lambda, \nu) = (0.2, 0.1, 1, 0.03, 1, 0)$.

The full equation and coefficients used to represent entire order parameter space of HfO_2 in Fig. 5b,c is:

$$\begin{aligned}H &= \beta_0 P_0{}^2 + \beta_1 Q_1{}^2 + \beta_2 Q_2{}^2 + \beta_3 Q_3{}^2 + \beta_4 Q_4{}^2 + \beta_5 Q_5{}^2 + \beta_6 Q_6{}^2 + \beta_{67} Q_6 Q_7 + \beta_7 Q_7{}^2 \\
&+ \gamma_{015} P_0 Q_1 Q_5 + \gamma_{024} P_0 Q_2 Q_4 + \gamma_{036} P_0 Q_3 Q_6 + \gamma_{037} P_0 Q_3 Q_7 \\
&+ \gamma_{123} Q_1 Q_2 Q_3 + \gamma_{146} Q_1 Q_4 Q_6 + \gamma_{147} Q_1 Q_4 Q_7 + \gamma_{256} Q_2 Q_5 Q_6 + \gamma_{257} Q_2 Q_5 Q_7 + \gamma_{345} Q_3 Q_4 Q_5 \\
&+ \delta_0 P_0{}^4 + \delta_{01} P_0{}^2 Q_1{}^2 + \delta_{02} P_0{}^2 Q_2{}^2 + \delta_{12} Q_1{}^2 Q_2{}^2 + \delta_{03} P_0{}^2 Q_3{}^2 + \delta_{13} Q_1{}^2 Q_3{}^2 + \delta_{23} Q_2{}^2 Q_3{}^2 + \delta_{04} P_0{}^2 Q_4{}^2 + \delta_{14} Q_1{}^2 Q_4{}^2 \\
&+ \delta_{24} Q_2{}^2 Q_4{}^2 + \delta_{34} Q_3{}^2 Q_4{}^2 + \delta_4 Q_4{}^4 + \delta_{05} P_0{}^2 Q_5{}^2 + \delta_{15} Q_1{}^2 Q_5{}^2 + \delta_{25} Q_2{}^2 Q_5{}^2 + \delta_{35} Q_3{}^2 Q_5{}^2 + \delta_{45} Q_4{}^2 Q_5{}^2 \\
&+ \delta_{06} P_0{}^2 Q_6{}^2 + \delta_{16} Q_1{}^2 Q_6{}^2 + \delta_{26} Q_2{}^2 Q_6{}^2 + \delta_{36} Q_3{}^2 Q_6{}^2 + \delta_{46} Q_4{}^2 Q_6{}^2 + \delta_{56} Q_5{}^2 Q_6{}^2 \\
&+ \delta_{067} P_0{}^2 Q_6 Q_7 + \delta_{167} Q_1{}^2 Q_6 Q_7 + \delta_{267} Q_2{}^2 Q_6 Q_7 + \delta_{367} Q_3{}^2 Q_6 Q_7 + \delta_{467} Q_4{}^2 Q_6 Q_7 + \delta_{567} Q_5{}^2 Q_6 Q_7 + \delta_{667} Q_6{}^3 Q_7 \\
&+ \delta_{67} Q_6{}^2 Q_7{}^2 + \delta_{07} P_0{}^2 Q_7{}^2 + \delta_{17} Q_1{}^2 Q_7{}^2 + \delta_{27} Q_2{}^2 Q_7{}^2 + \delta_{37} Q_3{}^2 Q_7{}^2 + \delta_{47} Q_4{}^2 Q_7{}^2 + \delta_{57} Q_5{}^2 Q_7{}^2 + \delta_{677} Q_6 Q_7{}^3 \\
&+ \delta_{0126} P_0 Q_1 Q_2 Q_6 + \delta_{0127} P_0 Q_1 Q_2 Q_7 + \delta_{0134} P_0 Q_1 Q_3 Q_4 + \delta_{0235} P_0 Q_2 Q_3 Q_5 + \delta_{0456} P_0 Q_4 Q_5 Q_6 + \delta_{0457} P_0 Q_4 Q_5 Q_7 \\
&+ \delta_{1245} Q_1 Q_2 Q_4 Q_5 + \delta_{1356} Q_1 Q_3 Q_5 Q_6 + \delta_{1357} Q_1 Q_3 Q_5 Q_7 + \delta_{2346} Q_2 Q_3 Q_4 Q_6 + \delta_{2347} Q_2 Q_3 Q_4 Q_7 \\
&- v(\lambda P_0 + \mu_{15} Q_1 Q_5 + \mu_{24} Q_2 Q_4 + \mu_{36} Q_3 Q_6 + \mu_{37} Q_3 Q_7)\end{aligned} \tag{S3}$$

Unlike others such as eq. 1, this equation does not include conventional factors of $1/n$ for the coefficients of Q^n terms. Fourth order terms $Q_1{}^4, Q_2{}^4, Q_3{}^4, Q_5{}^4, Q_6{}^4, Q_7{}^4$ which have minimal effect in the energy (Fig. S8) has been excluded from the full regression up to 4th order for better convergence in the P-P_0 plot.

DFT data used for regression involves combinations of following order parameters. Around I4/mmm: $P_0 = (-0.0707, 0, 0.0707)$ Å, $Q_1 = (-0.0707, 0, 0.0707)$ Å, $Q_2 = (-0.0717, 0, 0.0717)$ Å, $Q_3 = (-0.0717, 0, 0.0717)$ Å, $Q_4 = (-0.0717, 0, 0.0717)$ Å, $Q_5 = (-0.0152, 0, 0.0152)$ Å, $Q_6 = (-0.0254, 0, 0.0254)$ Å, $Q_7 = (-0.0215, 0, 0.0215)$ Å. Around $Pca2_1$: $P_0 = (0.3299, 0.4007, 0.4714)$ Å, $Q_1 = (0.3088, 0.3795, 0.4503)$ Å, $Q_2 = (0.3283, 0.4001, 0.4718)$ Å, $Q_3 = (0.3103, 0.3820, 0.4537)$ Å, $Q_4 = (-0.5411, -0.4694, -0.3977)$ Å, $Q_5 = (0.0708, 0.0860, 0.1013)$ Å, $Q_6 = (0.1344, 0.1597, 0.1851)$ Å, $Q_7 = (-0.1398, -0.1182, -0.0967)$ Å.

Derivation of hybrid-triggered instability with additional couplings

$$\begin{aligned}H &= \frac{\beta_0}{2} p_0{}^2 + \frac{\beta_1}{2} q_1{}^2 + \frac{\beta_2}{2} q_2{}^2 + \frac{\beta_3}{2} q_3{}^2 + \frac{\beta_4}{2} q_4{}^2 + \gamma_{012} p_0 q_1 q_2 + \gamma_{134} q_1 q_3 q_4 \\
&+ \frac{\delta_0}{4} p_0{}^4 + \frac{\delta_4}{4} q_4{}^4 + \frac{\delta_{12}}{2} q_1{}^2 q_2{}^2 + \frac{\delta_{13}}{2} q_1{}^2 q_3{}^2 - v(\lambda p_0)\end{aligned} \tag{S4}$$

Note that $\beta_4 < 0$, as q_4 corresponds to the order parameter of an unstable mode. The derivation follows the three steps presented in the main text. First, determine the partial derivatives.

$$\frac{\partial H}{\partial p_0} = \beta_0 p_0 + \gamma_{012} q_1 q_2 + \delta_0 p_0{}^3 - \lambda v = 0 \tag{S5}$$

$$\frac{\partial H}{\partial q_1} = \beta_1 q_1 + \gamma_{012} p_0 q_2 + \gamma_{134} q_3 q_4 + \delta_{12} q_1 q_2{}^2 + \delta_{13} q_1 q_3{}^2 = 0 \tag{S6}$$

$$\frac{\partial H}{\partial q_2} = \beta_2 q_2 + \gamma_{012} p_0 q_1 + \delta_{12} q_1{}^2 q_2 = 0 \tag{S7}$$

$$\frac{\partial H}{\partial q_3} = \beta_3 q_3 + \gamma_{134} q_1 q_4 + \delta_{13} q_1{}^2 q_3 = 0 \tag{S8}$$

$$\frac{\partial H}{\partial q_4} = \beta_4 q_4 + \gamma_{134} q_1 q_3 + \delta_4 q_4{}^3 = 0 \tag{S9}$$

Second, optimize q_i ($i \neq 1$).

$$q_2 = -\frac{\gamma_{012} p_0 q_1}{\beta_2 + \delta_{12} q_1{}^2} \tag{S10}$$

$$q_3 = -\frac{\gamma_{134} q_1 q_4}{\beta_3 + \delta_{13} q_1{}^2} = -\frac{\gamma_{134} q_1 \sqrt{-\frac{\beta_4}{\delta_4}}}{\beta_3 + \delta_{13} q_1{}^2} \tag{S11}$$

$$|q_4| = \sqrt{-\frac{\beta_4}{\delta_4}} \tag{S12}$$

Note that q_4 can be optimized for pre-trigger dielectric phase, as we are searching for $p_{0,c}$. Third, insert q_i back to $(\partial^2 H/\partial {q_1}^2)$ and determine the condition it is negative at $q_1 = 0$.

$$\left.\frac{\partial^2 H}{\partial {q_1}^2}\right|_{q_1=0} = \frac{\beta_1{\beta_2}^2{\beta_3}^2 - \beta_2{\beta_3}^2{\gamma_{012}}^2{p_0}^2 - {\beta_2}^2\beta_3{\gamma_{134}}^2{q_4}^2}{{\beta_2}^2{\beta_3}^2} < 0 \tag{S13}$$

$${p_0}^2 > \frac{\beta_1\beta_2\beta_3 - \beta_2{\gamma_{134}}^2{q_4}^2}{\beta_3{\gamma_{012}}^2} = \frac{\beta_1\beta_2 - \frac{\beta_2(-\beta_4){\gamma_{134}}^2}{\beta_3\delta_4}}{{\gamma_{012}}^2} \tag{S14}$$

$$|p_{0,c}| = \frac{\sqrt{\beta_1\beta_2 - \frac{\beta_2(-\beta_4){\gamma_{134}}^2}{\beta_3\delta_4}}}{|\gamma_{012}|} \tag{S15}$$

The numerator is reduced from $\sqrt{\beta_1\beta_2}$ to $\sqrt{\beta_1\beta_2 - \frac{\beta_2(-\beta_4){\gamma_{134}}^2}{\beta_3\delta_4}}$, so multiple coupling reduces the value of shared trigger. From Equation (S10) and (S11), simultaneous condensation of q_1, q_2 and q_3 is shown.

What we show is that with the additional trilinear coupling, instead of an additional trigger being created leading to second phase transition, the critical value $p_{0,\mathrm{c}}$ is decreased substantially and shared among both couplings—the simultaneous condensation of q_1, q_2, and q_3 occurs. In other words, the hybrid mode $q_1q_2q_3$ instability occurs at a lower p_0 compared to just q_1q_2 or q_1q_3.

Derivation of hybrid-triggered instability with polar hybrid mode

$$H = \frac{\beta_0}{2}{p_0}^2 + \frac{\beta_1}{2}{q_1}^2 + \frac{\beta_2}{2}{q_2}^2 + \gamma p_0q_1q_2 + \frac{\delta_0}{4}{p_0}^4 + \frac{\delta_{12}}{2}{q_1}^2{q_2}^2 - v(\lambda p_0 + \mu q_1q_2) \tag{S16}$$

First, determine the partial derivatives.

$$\frac{\partial H}{\partial p_0} = \beta_0p_0 + \gamma q_1q_2 + \delta_0{p_0}^3 - \lambda v = 0 \tag{S17}$$

$$\frac{\partial H}{\partial q_1} = \beta_1q_1 + \gamma p_0q_2 + \delta_{12}q_1{q_2}^2 - \mu vq_2 = 0 \tag{S18}$$

$$\frac{\partial H}{\partial q_2} = \beta_2q_2 + \gamma p_0q_1 + \delta_{12}{q_1}^2q_2 - \mu vq_1 = 0 \tag{S19}$$

Second, optimize q_i ($i \neq 1$).

$$q_2 = -\frac{(\gamma p_0 - \mu v)q_1}{\beta_2 + \delta_{12}{q_1}^2} \tag{S20}$$

Here, v also has to be represented as a function of p_0 and the coefficients. From Equation (S17):

$$v = \frac{\beta_0p_0 + \delta_0{p_0}^3}{\lambda} \tag{S21}$$

$$q_2 = -\frac{[(\gamma - \frac{\mu\beta_0}{\lambda})p_0 - \frac{\mu\delta_0}{\lambda}{p_0}^3]q_1}{\beta_2 + \delta_{12}{q_1}^2} \tag{S22}$$

Note that v was optimized for pre-trigger dielectric phase. Third, insert q_i back to $\partial^2 H/\partial {q_1}^2$ and determine the condition it is negative at $q_1 = 0$. The solution is equivalent to the derivation of eq. 1, where γp_0 is substituted to $\gamma p_0 - \mu v$.

$$\left.\frac{\partial^2 H}{\partial {q_1}^2}\right|_{q_1=0} = \frac{\beta_1{\beta_2}^2 - \beta_2(\gamma p_0 - \mu v)^2}{{\beta_2}^2} < 0 \tag{S23}$$

$$\left((\gamma - \frac{\mu\beta_0}{\lambda})p_0 - \frac{\mu\delta_0}{\lambda}{p_0}^3\right)^2 = \beta_1\beta_2 \tag{S24}$$

While the general solution cannot be expressed, when $\delta_0 \sim 0$:

$$|p_{0,c}| = \frac{\sqrt{\beta_1\beta_2}}{\left|\gamma - \frac{\mu\beta_0}{\lambda}\right|} \tag{S25}$$

Depending on the sign of μ, the polar hybrid mode can either increase or decrease $|p_{0,\mathrm{c}}|$. In the case of HfO_2, the direction of polarization from second order hybrid modes always oppose the direction of polarization from the polar mode. This would correspond to the same sign of γ and μ, which increases $|p_{0,\mathrm{c}}|$.

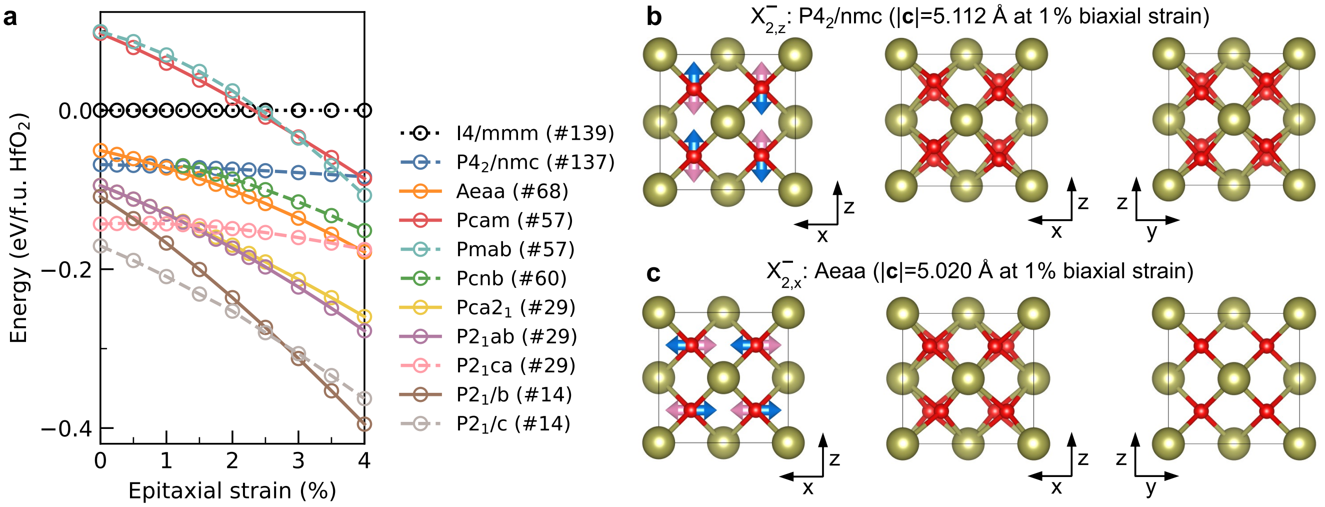

Figure S1. Biaxial strain phase diagram of HfO_2. a, Solid lines indicate phases derived from $X^-_{2,x}$, while dashed lines represent phases derived from $X^-_{2,z}$. **b,** Structural representation of $X^-_{2,z}$ mode and **c,** $X^-_{2,x}$ mode. Blue arrows represent oxygen displacements in the front half of the unit cell ($y = 0.75$ in direct coordinates), and the pink arrows represent those in the rear half ($y = 0.25$). In the absence of a monoclinic lattice transformation (as well as Pbca or domain-separated structures from ref.[9]), the Aeaa phase emerges as the most stable nonpolar ground state under moderate tensile strain, derived from structural instability in the fluorite HfO_2. Experimental observations of the lattice constants from[10,11] further support this. Transition from $Pca2_1$ to $P4_2/nmc$ increases the interplanar spacing, whereas transition from $Pca2_1$ to Aeaa reduces it. (c = 5.038 Å for $Pca2_1$ phase)

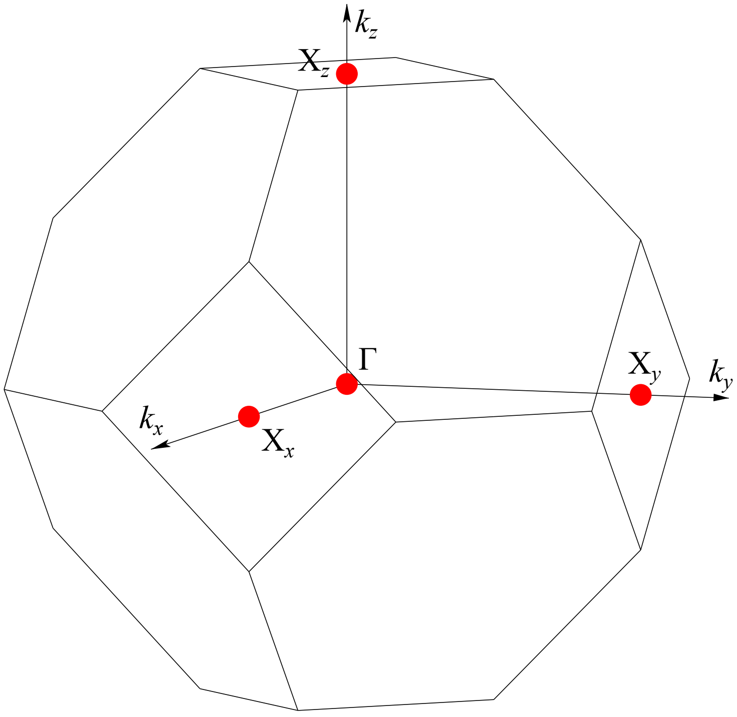

Figure S2. First Brillouin zone of a face-centered cubic lattice system[3]. The coordinates of the wavevectors $\boldsymbol{k}_{X_x}$, $\boldsymbol{k}_{X_y}$, and $\boldsymbol{k}_{X_z}$ corresponding to the positions of the X_x, X_y, and X_z points in reciprocal space, are $(2\pi/a, 0, 0)$, $(0, 2\pi/a, 0)$, and $(0, 0, 2\pi/a)$ respectively in (k_x, k_y, k_z). The sum $\boldsymbol{k}_{X_x} + \boldsymbol{k}_{X_y} + \boldsymbol{k}_{X_z}$ forms a Γ-point modulo in reciprocal space, enabling a trilinear coupling with irrep equal to that of the energy (Γ_1^+). Note that the sum of wavevectors being equal to a Γ-point modulo is a necessary condition for irrep equivalence with the energy, and not a sufficient one. For instance, the wavevector sum arising from the bilinear coupling $Q_1 Q_5$ ($\boldsymbol{k}_{X_x} + \boldsymbol{k}_{X_x}$) also forms a Γ-point modulo but exhibits irrep equivalent to polarization (Γ_4^-). Consequently, the bilinear coupling does not directly appear in the energy expression.

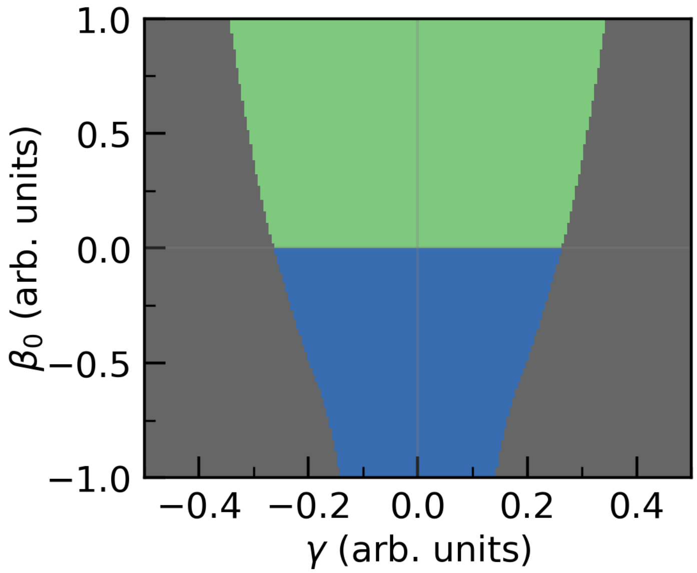

Figure S3. Phase diagram of a hybrid-triggered ferroelectric by eq. 1, by second-order p_0 coefficient β_0 and trilinear coupling coefficient γ. Green area represent the nonpolar ground state where $p_0 = q_1 = q_2 = 0$, blue area the proper ferroelectric ground state where $p_0 \neq 0$, $q_1 = q_2 = 0$, and the gray area the hybrid-triggered polar ground state where $p_0, q_1, q_2 \neq 0$. The hybrid-triggered ground state exists for cases when the polar mode is both soft and hard, if the trilinear coupling is strong enough. Similar phase diagram can be found for a hypothetical case of quadratic-linear order parameter coupling[12], and charge density wave order in kagome metals involving a trilinear coupling[13]. The tricritical points are along $\beta_0 = 0$ for all phase diagrams. In the cases where the components of hybrid mode remain hard when $\beta_0 = 0$, there is a tricritical point for each sign of non-zero value of γ. In the case of kagome metals where all three components of trilinear coupling have the same second-order coefficient, the two tricritical points merge into one point at $\gamma = 0$.

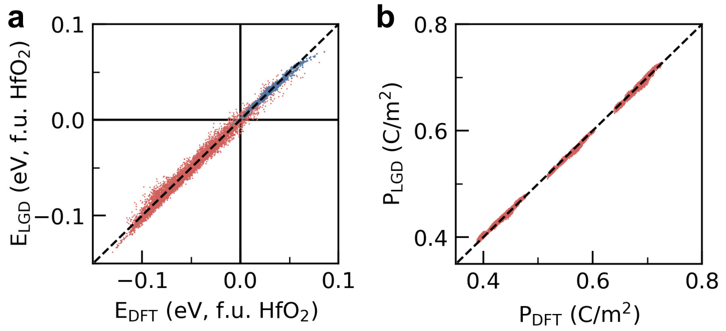

Figure S4. Symmetry-adapted regression of DFT data using LGD theory a, Energy and **b,** polarization data of HfO_2 under 1% tensile biaxial strain. Blue points correspond to 3^8 data points around the high-symmetry I4/mmm phase, while red points correspond to 3^8 data points around the ferroelectric Pca2_1 phase.

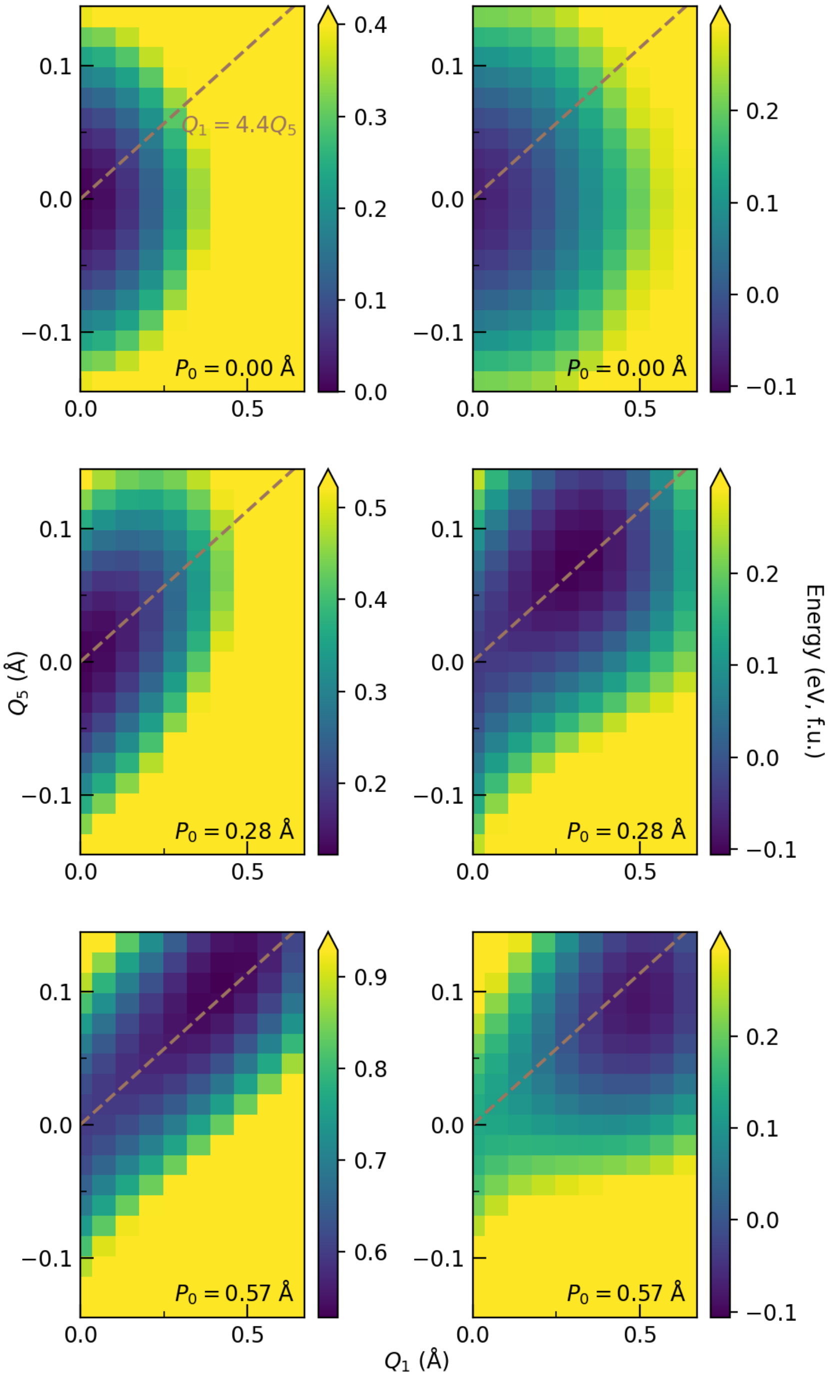

Figure S5. Two-dimensional DFT-calculated energies of HfO_2, illustrating hybrid-triggered ferroelectricity and $P_0Q_1Q_5$ coupling. The results compare scenarios where all other order parameters are fixed to zero (left) and fully relaxed (right). One-dimensional data shown in Fig. 2c is along $Q_1 = 4.4Q_5$ line which preserve the ratio at the ferroelectric minimum. The fixed ratio is not necessarily imposed from the hybrid-triggered ferroelectricity. Note that even when the hybrid mode Q_1Q_5 instability appears, individual modes Q_1 and Q_5 can remain hard.

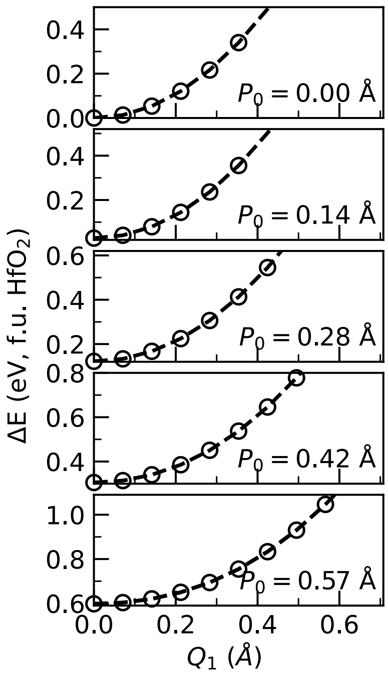

Figure S6. Biquadratic coupling between P_0 and Q_1. A slight negative biquadratic coupling exists between P_0 and Q_1[14]. However, in contrast to the trilinear and quadlinear couplings shown in Fig. 2, this coupling between the two hard modes does not induce a new local minimum or phase transition. The negative biquadratic coupling also occurs for ${Q_1}^2{Q_4}^2$, ${Q_3}^2{Q_4}^2$, ${P_0}^2{Q_5}^2$, ${Q_3}^2{Q_5}^2$, ${Q_1}^2{Q_6}^2$, ${Q_1}^2{Q_7}^2$, and ${Q_4}^2{Q_7}^2$ without major contribution to energy.

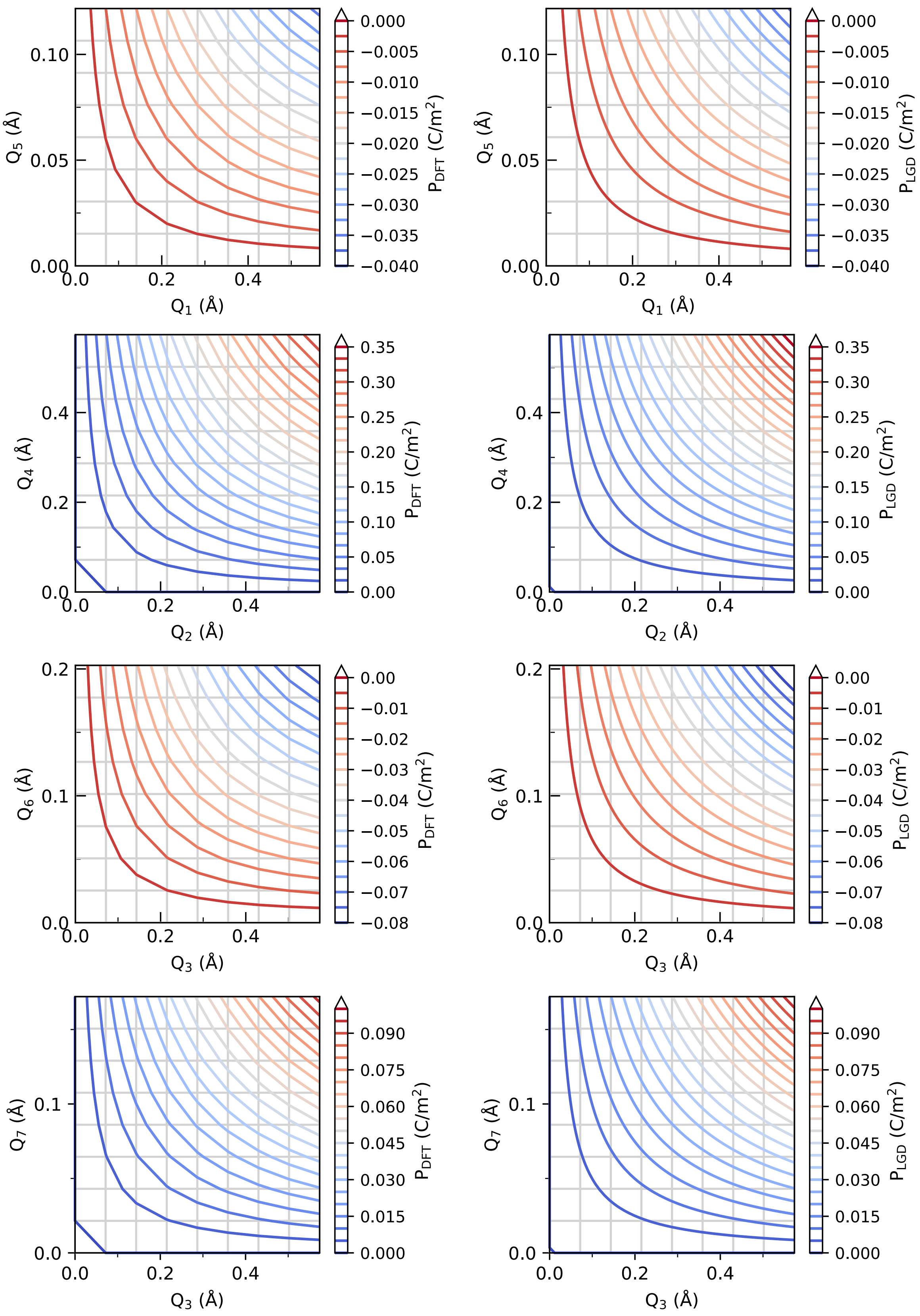

Figure S7. Polarization from hybrid nonpolar modes. Polarization of 1% biaxial strained HfO_2 from DFT calculations (left) and symmetry-guided LGD theory up to second order (right).

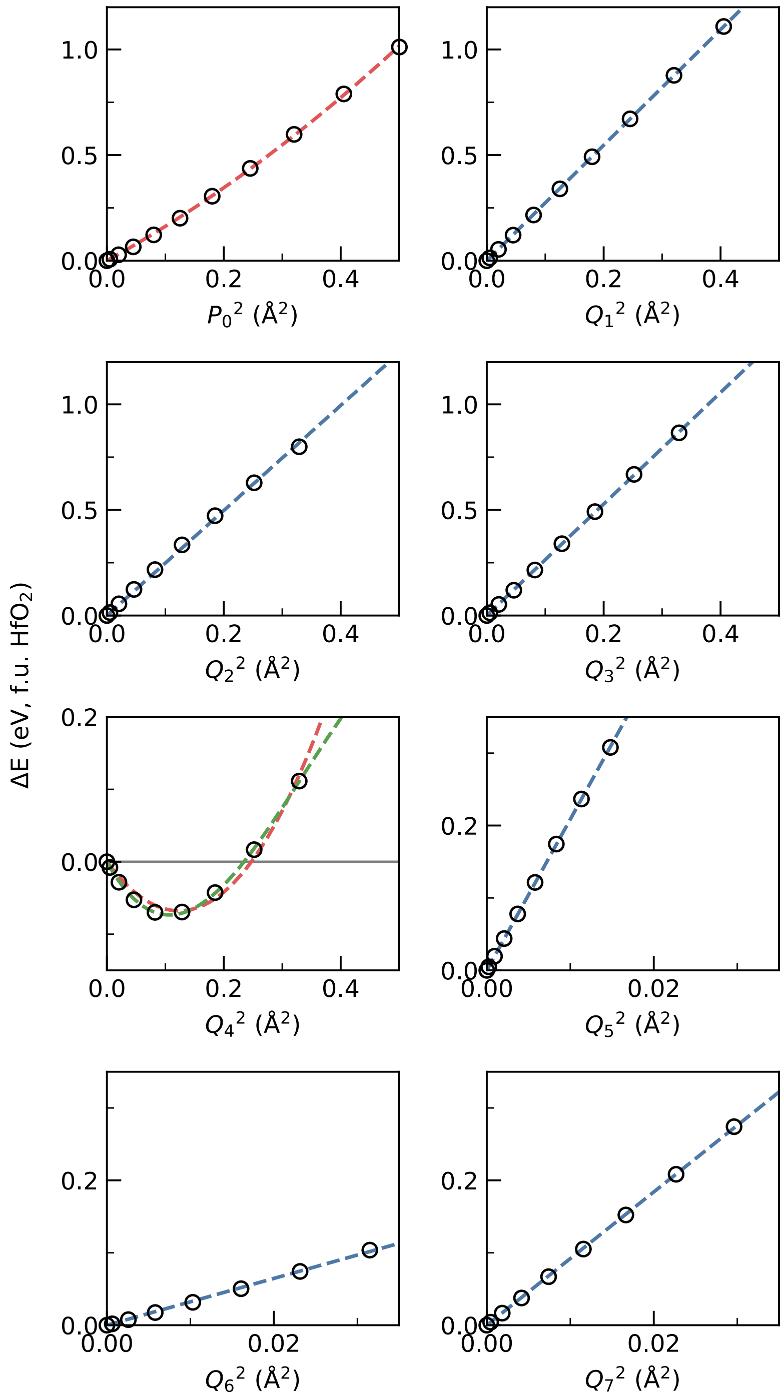

Figure S8. Contribution of each mode to energy from DFT. Blue, red and green dotted lines each represent 1st, 2nd and 3rd order regression of energy with respect to Q^2 through the origin. Linear regression fits most order parameters squared, except P_0 and Q_4.

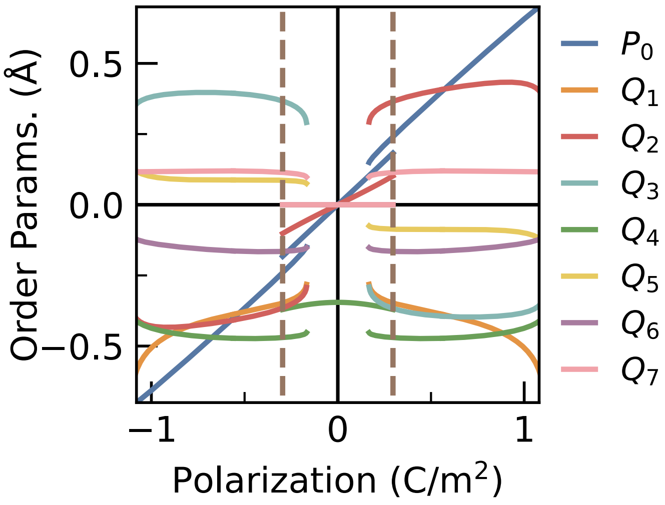

Figure S9. Alternative domain switching of HfO_2. Despite the presence of 8 modes, each triggered phase only have 2 domains that share the same sign of Q_4. The domains not shown in Fig. 5B is displayed here. There is equal chance of the modes condensing to each domain when the ferroelectricity is triggered.

Table S1. Irreducible representation (irrep) of order parameters from different parent structures. The displacements for Hf ion at $(0,0,0)$, the O_1 ion at $(0.25, 0.25, 0.25)$, and the O_2 ion at $(0.75, 0.25, 0.25)$, given in direct coordinates of the high-symmetry structure are given as reference positions for defining the ionic displacements. The remaining atomic displacements are subsequently determined by the corresponding wavevector. Note that Cartesian bases follow the conventional lattice of $Fm\bar{3}m$—conventional lattice transformation from $Fm\bar{3}m$ to I4/mmm requires rotation of Cartesian bases around z axis by $\pi/4$.

Order Parameter	Irrep		Ionic displacements			Space Group
	$Fm\bar{3}m$	I4/mmm	Hf	O1	O2	
η_{zz}	$\Gamma_1^+ \oplus \Gamma_3^+$	Γ_1^+	-	-	-	I4/mmm (#139)
P_0	Γ_4^-	Γ_3^-	-	$-u_z$	$-u_z$	I4mm (#107)
Q_1	$X_{5,y}^+$	$X_{3,y}^+$	-	u_z	u_z	Bmeb (#64)
Q_2	$X_{5,x}^+$	$X_{2,x}^+$	-	u_y	$-u_y$	Aeam (#64)
Q_3	$X_{5,z}^+$	M_5^+	-	u_x	u_x	Ccme (#64)
Q_4	$X_{2,x}^-$	$X_{1,x}^-$	-	u_x	u_x	Aeaa (#68)
Q_5	$X_{3,y}^-$	$X_{4,y}^-$	u_y	-	-	Bmem (#67)
Q_6	$X_{5,z}^-$	M_5^-	u_x	-	-	Ccmm (#63)
Q_7	$X_{5,z}^-$	M_5^-	-	u_y	$-u_y$	Ccmm (#63)

Table S2. Inversion breaking of the symmetry-modes. i_1 to i_4 denote the inversion centers present in the high-symmetry conventional fluorite unit cell. For every inversion center (x,y,z), there are 7 more translation induced inversion centers at $(x+0.5, y, z)$, $(x, y+0.5, z)$, $(x, y, z+0.5)$, $(x, y+0.5, z+0.5)$, $(x+0.5, y, z+0.5)$, $(x+0.5, y+0.5, z)$, and $(x+0.5, y+0.5, z+0.5)$. Symbol ○ indicates that the inversion center is preserved upon the condensation of the mode, while × signifies that the inversion center is destroyed.

Inversion center	Direct coordinates			η_{zz}	P_0	Q_1	Q_2	Q_3	Q_4	Q_5	Q_6	Q_7
	x	y	z									
i_1	0	0	0	○	×	○	○	○	×	×	×	×
i_2	0	0.25	0.25	○	×	×	○	×	×	○	○	○
i_3	0.25	0	0.25	○	×	○	×	×	○	×	○	○
i_4	0.25	0.25	0	○	×	×	×	○	○	○	×	×

References

1. Jung, S. & Birol, T. Octahedral-rotation-induced, antiferroelectric-like double hysteresis in strained perovskites. *Nano Letters* (2025).
2. King-Smith, R. & Vanderbilt, D. Theory of polarization of crystalline solids. *Physical Review B* **47,** 1651 (1993).
3. Aroyo, M. I. *et al.* Crystallography online: Bilbao crystallographic server. *Bulg. Chem. Commun* **43,** 183–197 (2011).
4. Hatch, D. M. & Stokes, H. T. INVARIANTS: program for obtaining a list of invariant polynomials of the order-parameter components associated with irreducible representations of a space group. *Journal of applied crystallography* **36,** 951–952 (2003).
5. Stokes, H. T. & Hatch, D. M. FINDSYM: program for identifying the space-group symmetry of a crystal. *Journal of Applied Crystallography* **38,** 237–238 (2005).

6. Campbell, B. J., Stokes, H. T., Tanner, D. E. & Hatch, D. M. ISODISPLACE: a web-based tool for exploring structural distortions. *Journal of Applied Crystallography* **39,** 607–614 (2006).

7. Stokes, H. T., Campbell, B. J. & Hatch, D. M. *ISOTROPY Software Suite* https://iso.byu.edu.

8. Cowley, R. Structural phase transitions I. Landau theory. *Advances in physics* **29,** 1–110 (1980).

9. Lee, H.-J. *et al.* Scale-free ferroelectricity induced by flat phonon bands in HfO_2. *Science* **369,** 1343–1347 (2020).

10. Cheema, S. S. *et al.* Enhanced ferroelectricity in ultrathin films grown directly on silicon. *Nature* **580,** 478–482 (2020).

11. Cheema, S. S. *et al.* Emergent ferroelectricity in subnanometer binary oxide films on silicon. *Science* **376,** 648–652 (2022).

12. Gufan, Y. M. & Torgashev, V. Phenomenological theory of changes in multicomponent order parameters. *Soviet Physics, Solid State* **22,** 951–956 (1980).

13. Christensen, M. H., Birol, T., Andersen, B. M. & Fernandes, R. M. Theory of the charge density wave in AV_3Sb_5 kagome metals. *Physical Review B* **104,** 214513 (2021).

14. Zhou, S., Zhang, J. & Rappe, A. M. Strain-induced antipolar phase in hafnia stabilizes robust thin-film ferroelectricity. *Science Advances* **8,** eadd5953 (2022).
